# Acetylation of HDAC1 and degradation of SIRT1 form a positive feedback loop to regulate p53 acetylation during heat-shock stress

**DOI:** 10.1038/cddis.2015.106

**Published:** 2015-05-07

**Authors:** H Yang, B Yan, D Liao, S Huang, Y Qiu

**Affiliations:** 1 Department of Anatomy and Cell Biology, College of Medicine, University of Florida, Gainsville, FL 32610, USA; 2 Department of Biochemistry and Molecular Biology, College of Medicine, University of Florida, Gainesville, FL 32610, USA

## Abstract

The tumor suppressor p53 is an essential transcription factor that sensitively regulates cellular responses to various stresses. Acetylation, a critically important posttranslational modification of p53, is induced in response to cellular stresses. P53 acetylation level strongly correlates with protein stability and activity. The steady-state level of p53 acetylation is balanced by dynamic acetylation and deacetylation. Despite the function of p53 acetylation being well studied, how the steady state of p53 acetylation level is regulated in response to cellular stresses remains unclear. In particular, the dynamic regulation of the deacetylase activities responsible for p53 deacetylation during cellular stress is unknown. In the current study, we investigated the dynamic regulation of HDAC1 (histone deacetylase 1) and SIRT1 (sirtuin 1), two major enzymes for p53 deacetylation, during cell stress. We found that various cell stress events induce HDAC1 acetylation. The increased level of HDAC1 acetylation correlates with the level of p53 acetylation. Acetylated HDAC1 loses the ability to deacetylate p53. Cellular stresses also promote the decline of the SIRT1 protein in a proteasome-dependent pathway, which also results in the increase of p53 acetylation. Importantly, the decreased level of SIRT1 also contributes to the accumulation of HDAC1 acetylation as SIRT1 deacetylates HDAC1. Therefore, the increase of HDAC1 acetylation and reduced level of SIRT1 protein during cellular stress directly link to the induction of p53 acetylation. These results unveil the mechanism underlying the dynamic regulation of p53 acetylation during cell stress.

Reversible acetylation and deacetylation of lysine residues present in histones have long been implicated in the regulation of transcription. A recent *in vivo* acetylome study unveils that more than a thousand non-histone proteins can be dynamically acetylated upon the treatment of a histone deacetylase (HDAC) inhibitor, suggesting that acetylation has a key role in the regulation of virtually all cellular processes.^1^ Mammalian HDACs are divided into four classes (I, II, III and IV) based on the sequence homology to the yeast histone deacetylases Rpd3 (reduced potassium dependency 3), Hda1 (histone deacetylase 1), and Sir2 (silent information regulator 2 or sirtuin), respectively.^2^ Class I HDACs include HDAC 1, 2, 3 and 8; class II HDACs contain HDACs 4, 5, 6, 7, 9 and 10, whereas class III enzymes require the coenzyme NAD+ as a cofactor. Class IV contains HDAC11. In mammals, seven sirtuin proteins (SIRT1–7) have been found.^3^ SIRT1, a proto member of the sirtuin family, deacetylates histone and many non-histone proteins that are involved in many aspects of cellular function, including cell growth, apoptosis, aging, calorie restriction and tumorigenesis.^4, 5^ Although the precise cellular functions of the different HDAC enzymes are still poorly understood, evidence suggests that different members of the HDAC family have distinct functions.^6, 7^ HDACs undergo various posttranslational modifications, such as phosphorylation, sumoylation, ubiquitination, S-nitrosylation and acetylation,^8, 9, 10, 11, 12^ which modulate enzymatic activity, protein stability and their interactions with other proteins. We recently reported that HDAC1 can be acetylated after the induction of a transcription program.^11, 13, 14^ Acetylated HDAC1 not only loses its own histone deacetylase activity but also transrepresses the deacetylase activity of HDAC2.^15^ Interestingly, acetylated HDAC1 can be reversibly deacetylated by SIRT1.^13^ Therefore, dynamic acetylation and deacetylation of HDAC1 can ultimately regulate HDAC1 deacetylase activity during cellular events.

P53 is a key transcription factor that is activated in response to various cellular stresses. P53 regulates the expression of a large number of target genes.^16^ Through the activation of target genes, p53 induces cell-cycle arrest, cell death and senescence. One of the first identified transcriptional targets of p53 is the cyclin-dependent kinase (CDK) inhibitor p21^Waf1/Cip1^.^17^ CDKs have an important role in regulating cell-cycle progression, and the inhibition of CDK activity by p21^Waf1/Cip1^ results in a cell-cycle arrest.^18^ The p53 protein level rises markedly within minutes of cellular stress treatment. This is achieved through posttranslational modifications of the p53 polypeptide, while there is no marked induction of p53 mRNA levels after DNA damage or other stress.^19, 20^ This provides a particularly rapid, sensitive, flexible and readily reversible mechanism for p53 activity regulation in response to a number of different cellular stresses. P53 was the first non-histone protein shown to be acetylated.^21^ Nine acetylation sites have been identified in p53. The histone acetyltransferases (HATs) responsible for these modifications include p300/CBP, p300/CBP-associated factor (pCAF) and the MYST family HATs.^22, 23, 24^ Six lysine residues in the C-terminal regulatory domain are acetylated by CBP/p300.^23^ Acetylation of p53 activates its sequence-specific DNA binding and its transcriptional activity, as well as enhances the stability of the p53 protein, owing to the mutual exclusion of acetylation and ubiquitination,^25^ therefore, resulting in p53-dependent gene activation in response to cellular stress.^26^

The steady-state level of p53 acetylation is maintained by the balance of dynamic acetylation and deacetylation (reviewed in refs ^26, 27, 28, 29^). P53 can be deacetylated by HDAC1. The physical interaction of HDAC1 reduces the steady-state levels of acetylated p53 and inhibits p53-dependent transcriptional activation, cell growth arrest and apoptosis.^30^ In addition, p53 can also be deacetylated by SIRT1.^31, 32^ SIRT1 preferentially deacetylates p53 at the K382 acetylation site and has a profound negative impact on the capacity of p53 to induce the expression of target genes involved in apoptosis.^31, 33^ The inhibition of SIRT1 by a specific inhibitor causes p53 hyperacetylation and increases p53-dependent transcriptional activity.^34^ In the current study, we uncovered the mechanism underlying the induction of p53 acetylation upon cellular stresses. We found that the downregulation of deacetylase activity has a key role in this process. Our data show that various cell stresses induce HDAC1 acetylation, which is catalyzed by p300. After acetylation, HDAC1 is no longer able to deacetylate p53. Cellular stresses also affect SIRT1 protein stability, which also results in the increase of p53 acetylation. Importantly, the decrease of SIRT1 also increases HDAC1 acetylation as SIRT1 deacetylates HDAC1. Therefore, these results unveil the mechanism underlying the regulation of p53 acetylation during cell stresses.

## Results

### HDAC1 acetylation is induced under cellular stresses

**Figure 1:**
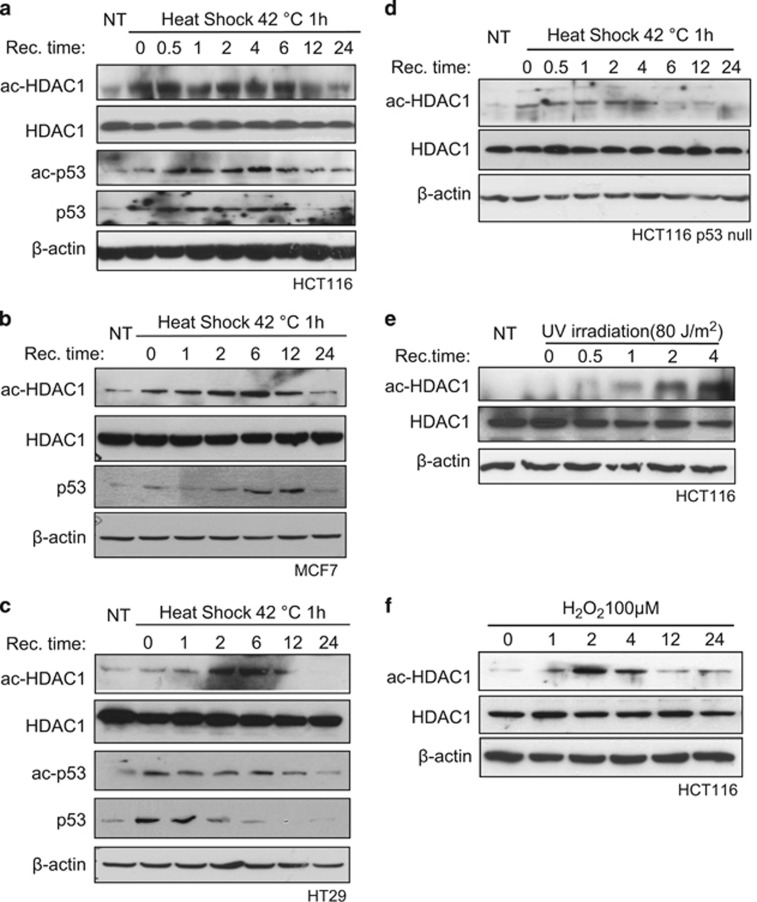
Acetylated HDAC1 increased during various cellular stresses. (**a**) HCT116 cells were treated at 42 °C for 1 h and then recovered at 37 °C for an indicated time period. The cell extracts were harvested and tested for protein levels with indicated antibodies. *β*-Actin was used as the loading control. MCF-7 cells (**b**) and HT-29 (**c**) cells were subjected to heat shock at 42 °C for 1 h and then recovered for an indicated time. The cell extracts were harvested and tested for protein levels with indicated antibodies. (**d**) HCT116 p53-null cells were treated at 42 °C for 1 h and then recovered at 37 °C for an indicated time period. The cell extracts were harvested and tested for protein levels with indicated antibodies. *β*-Actin was used as the loading control. (**e**) HCT116 cells were exposed to UV irradiation (80 J/m^2^) and recovered at an indicated time. The cell extracts were harvested and tested for protein levels with indicated antibodies.(**f**) HCT116 cells were incubated with DMEM medium containing 100 *μ*M H_2_O_2_ for an indicated time. The cells were harvested and subjected to western blot

Previous studies showed that HDAC1 acetylation modulates its deacetylase activity and can be induced during differentiation events and upon the induction of DNA double-strand break.^11, 13, 14, 15^ We therefore investigated whether various cellular stresses can also influence HDAC1 acetylation levels. HCT116 cells were subjected to 42 °C heat shock for 1 h. HDAC1 acetylation is induced after the treatment (Figure 1a). The acetylation level remains elevated until 12 h after treatment. Heat shock also induces HDAC1 acetylation in other cell lines, such as MCF-7 and HT-29 cells (Figures 1b and c), indicating that HDAC1 acetylation can be rapidly induced under heat-shock stress in various cells.

It is well documented that various cellular stress can induce rapid p53 acetylation, which in return stabilizes p53 protein.^20, 31, 35, 36, 37, 38^ We investigated whether p53 protein level correlates with HDAC1 acetylation. P53 protein level increases right after heat-shock treatment and decreases at the same time when HDAC1 acetylation levels started to reduce in all cell lines we tested (Figures 1a–c). Interestingly, p53 acetylation also follows HDAC1 acetylation pattern, suggesting that HDAC1 acetylation may attenuate p53 deacetylation. We then determined whether p53 is required for HDAC1 acetylation. The result shows that HDAC1 acetylation is also induced in the absence of p53 in HCT116 p53-null cells (Figure 1d), suggesting that the induction of HDAC1 acetylation by heat shock is p53 independent.

Next, we investigated whether other types of cellular stress can also induce HDAC1 acetylation. HCT116 cells were treated under UV irradiation, and HDAC1 acetylation is induced about 1 h after UV treatment (Figure 1e). Similarly, H_2_O_2_ treatment also elevates HDAC1 acetylation 1 h after treatment (Figure 1f). Collectively, induction of HDAC1 acetylation may be a general event for cellular stress response.

### Acetylated HDAC1 fails to deacetylate p53

**Figure 2:**
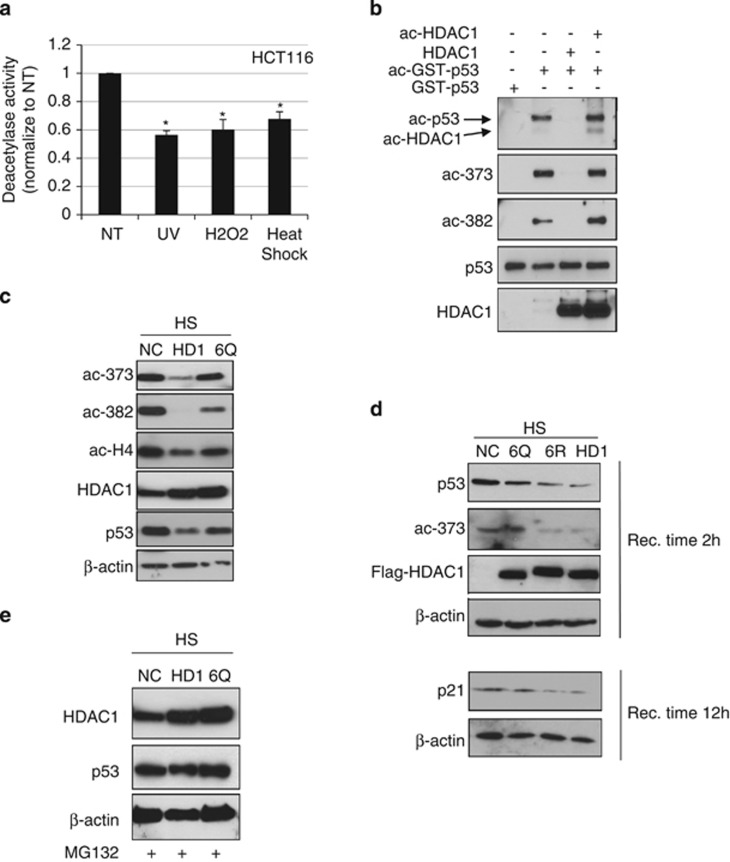
Acetylated HDAC1 does not deacetylate p53. (**a**) The HCT116 cells were treated with various stresses, HDAC1 proteins were immunoprecipitated and the bound proteins were subjected to deacetylation assay. For UV treatment, cells were exposed to 80 J/m^2^ UV. For H_2_O_2_ treatment, cells were incubated with 100 *μ*M H_2_O_2_ for 2 h. For heat-shock treatment, cells were treated at 42 °C for 1 h and then recovered at 37 °C for 2 h. The deacetylase activities were normalized to non-treatment (NT) control (mean±S.E.). *Significant difference compared with the non-treatment control (*P*<0.05 by Student's *t*-test). (**b**) Flag-HDAC1 and GST-p53 were acetylated by p300 *in vitro*. The acetylated p53 is then deacetylated by HDAC1 or ac-HDAC1. The reaction mix is subjected to western blot with indicated antibodies. (**c**) HCT116 cells were transfected with wild-type HDAC1 or ac-HDAC1 mimic mutant 6Q. The cells were treated with heat shock at 42 °C for 1 h and then recovered for 2 h. (**d**) HCT116 cells were infected with HDAC1, HDAC1 6R or HDAC1 6Q expressing retrovirus. The stable cells were treated with heat shock at 42 °C for 1 h and then recovered for 2 or 12 h as indicated. The cells were then harvested and subjected to western blot with indicated antibodies. (**e**) HCT116 cells were treated with 10 *μ*M MG132 for 2 h, followed by heat shock at 42 °C for 1 h, and then recovered for 2 h. The cell extracts were harvested and subjected to western blot with indicated antibodies

It has been shown that HDAC1 acetylation attenuates its deacetylase activity.^11, 15^ We therefore examined whether stress-induced HDAC1 acetylation affects HDAC1-associated deacetylase activity. HDAC1 was immunoprecipitated and histone deacetylase activity was tested. The result showed that various stress treatments reduce HDAC1-associated histone deacetylase activity (Figure 2a). Therefore, stress induces HDAC1 acetylation and results in the reduction of overall HDAC1-associated histone deacetylase activity.

As HDAC1 is a major deacetylase for p53 and HDAC1 acetylation correlates with p53 acetylation pattern (Figures 1a–c), we performed *in vitro* deacetylation assay to test whether acetylated HDAC1 affects p53 deacetylation. *In vitro* acetylated p53 was incubated with wild-type HDAC1 or *in vitro* acetylated HDAC1. The acetylation levels on two p53 acetylation sites were examined. These two sites have been reported to be deacetylated by HDAC1.^39^ The result shows that acetylated HDAC1 is no longer able to deacetylate p53 at both sites (Figure 2b). Next, we tested whether acetylated HDAC1 is also unable to deacetylate p53 *in vivo*. HDAC1 or HDAC1 6Q, which has six acetylated lysine mutated to glutamine to mimic acetylated HDAC1,^11^ was transfected into HCT116 cells and cells were subjected to heat shock. The wild-type HDAC1 was able to deacetylate p53 on both acetylation sites; however, acetyl-mimic HDAC1 has less activity to deacetylate p53, as well as histone H4 (Figure 2c). Therefore, acetylated HDAC1 deacetylates p53 to a lesser extent than p53 deacetylated by wild-type HDAC1 *in vitro* and *in vivo*. To confirm the transient transfection results, we stably expressed Flag-tagged HDAC1, acetyl-mimic HDAC1 6Q or non-acetyl-mimicking HDAC1 6R in HCT116 cells. We found that consistent with results from transient transfection experiments, overexpression of HDAC1 or HDAC1 6R reduces heat-shock-induced p53 acetylation and p53 protein level (Figure 2d). However, the overexpression of acetyl-mimic HDAC1 does not affect p53 acetylation, suggesting HDAC1 acetylation affects p53 acetylation level. We also measured the p21 protein level on those cell lines, it shows that heat shock induced p21 expression is largely affected by overexpression of HDAC1 or non-acetyl-mimicking HDAC1 but not acetyl-mimicking HDAC1 (Figure 2d). Therefore, our study shows that acetylation of HDAC1 after heat shock is a key step for activation of p53 and its downstream targets.

Interestingly, p53 protein levels are reduced in cells overexpressed HDAC1 (Figures 2c and d). We speculate that the decrease of p53 acetylation results in p53 instability. To test this, cells overexpressed HDAC1 or HDAC1 6Q were treated with the proteasome inhibitor MG132 and subsequently treated with heat shock. P53 levels were significantly rescued (Figure 2e) compared with cells without MG132 treatment (Figures 2c and d). Therefore, our result shows that heat-shock stress induces HDAC1 acetylation and, consequently, acetylated HDAC1 fails to deacetylate p53, resulting in the increase of p53 acetylation and p53 protein stability.

### P300 is a major acetyltransferase for HDAC1 acetylation *in vivo*

**Figure 3:**
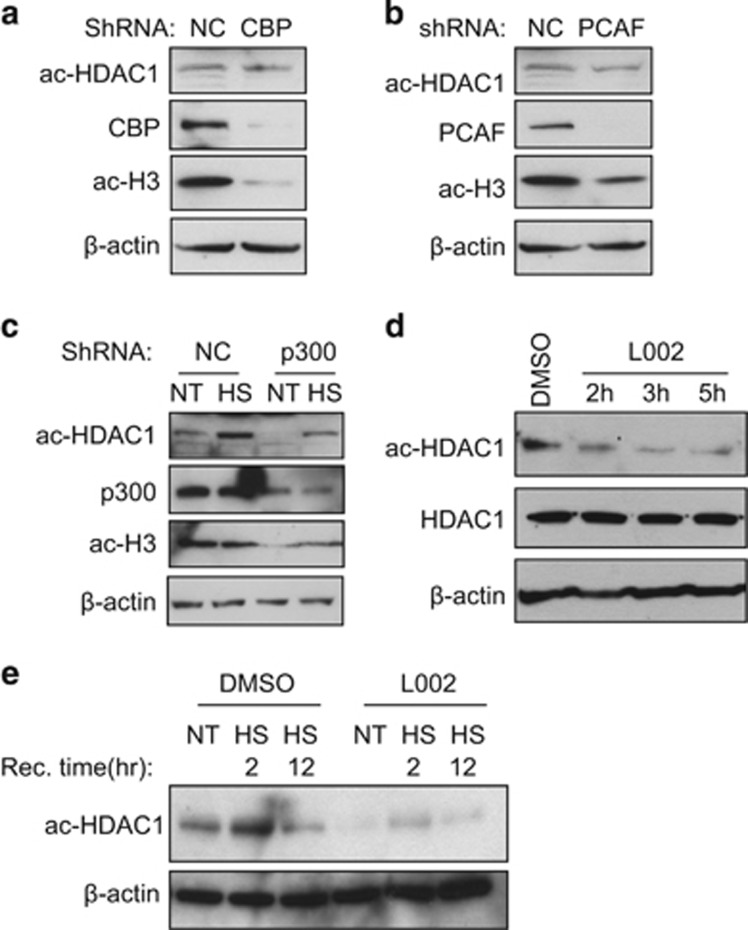
p300 but not other HAT can acetylate HDAC1 during heat shock. (**a** and **b**) The acetyltransferase CBP (**a**) and PCAF (**b**) were knocked down with shRNAs in HCT116 cells. The cell extracts were harvested and subjected to western blot with indicated antibodies. (**c**) p300 was knocked down with shRNA in HCT116 cells, which was subjected to heat shock at 42 °C for 1 h and recovered at 37 °C for 2 h. The cell extracts were harvested and subjected to western blot with indicated antibodies. (**d**) HCT116 cells were treated by p300 inhibitor L002 (10 *μ*M) for indicated time period and the cell extracts were subjected to western blot with indicated antibodies. (**e**) HCT116 cells were treated by p300 inhibitor L002 (10 *μ*M) for 5 h, subjected to 42 °C heat shock for 1 h and then recovered for 2 or 12 h. The cell extracts were harvested and subjected to western blot with indicated antibodies

Although it is well documented that HDAC1 can be acetylated, the acetyltransferease that is responsible for its acetylation *in vivo* was never investigated. We, therefore, knocked down each acetyltransferase to determine which enzyme acetylates HDAC1. We found that CBP or PCAF knockdown does not affect HDAC1 acetylation in HCT116 cells (Figures 3a and b). However, knocking down p300 significantly reduces HDAC1 acetylation levels both under normal and heat-shock conditions (Figure 3c). As p300 knockdown is not efficient and p300 knockdown also affects HDAC1 protein level,^40^ we also tested whether a p300 inhibitor reduces HDAC1 acetylation levels. The p300 inhibitor L002^41^ efficiently inhibited HDAC1 acetylation under both control and heat-shock conditions without affecting HDAC1 protein level (Figures 3d and e). This result indicates that p300 is responsible for HDAC1 acetylation *in vivo*.

### The reduction of SIRT1 after heat shock mediates the increase of HDAC1 acetylation

**Figure 4:**
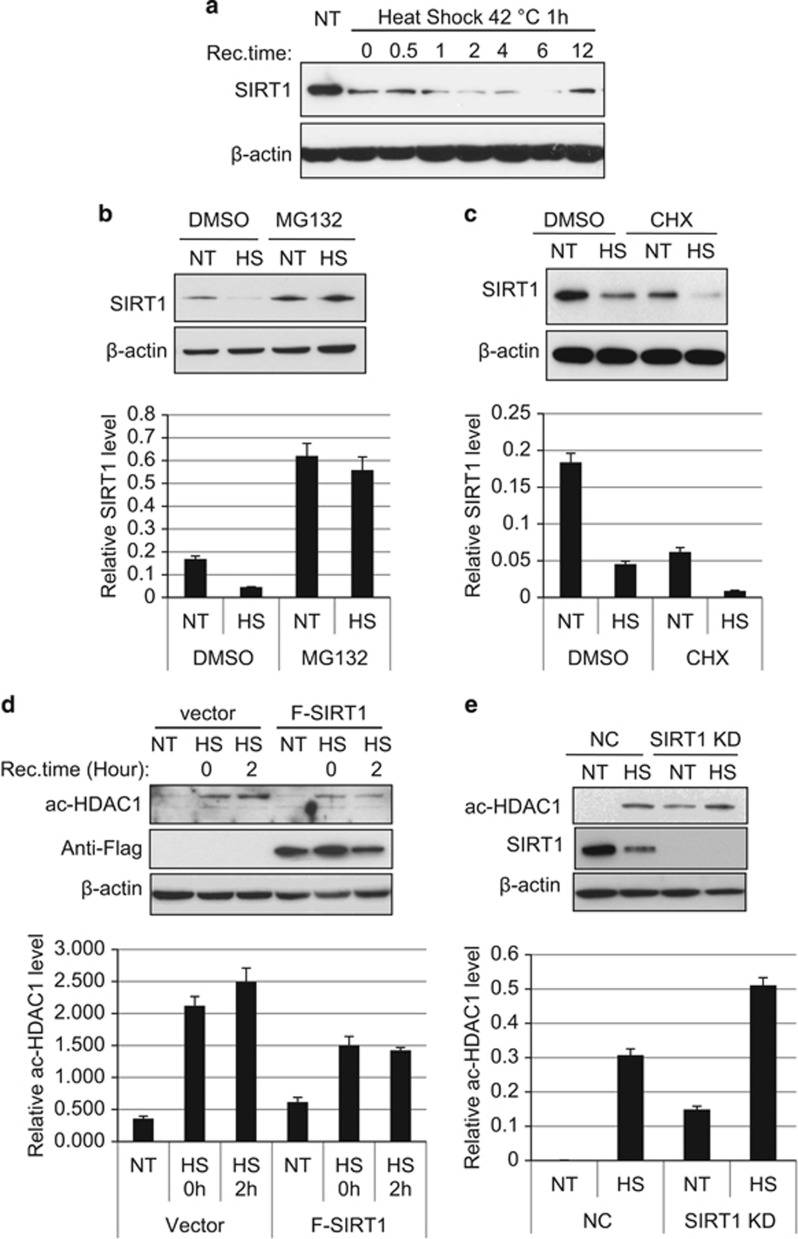
SIRT1-mediated HDAC1 deacetylation decreases during heat shock. (**a**) HCT116 cells were subjected to heat shock at 42 °C for 1 h and then recovered at 37 °C for an indicated time. SIRT1 protein level was tested by western blot. (**b**) HCT116 cells were treated with or without 10 *μ*M MG132 for 2 h and then subjected to heat shock. Cells were recovered at 37 °C for 2 h. Upper panel shows representative western blots and lower panel shows quantification of SIRT1 levels normalized to *β*-actin controls. (**c**) HCT116 cells were treated with or without 10 *μ*M CHX for 2 h and then subjected to heat shock. Cells were rescued at 37 °C for 2 h. Upper panel shows representative western blots and lower panel shows quantification of SIRT1 levels normalized to *β*-actin controls. (**d**) Flag-SIRT1 was overexpressed in HCT116 cells and then the cells were subjected to heat shock at 42 °C for 1 h and recovered for indicated times. The cell extracts were harvested and subjected to western blot with indicated antibodies. Upper panel shows representative western blots and lower panel shows quantification of ac-HDAC1 levels normalized to *β*-actin controls. (**e**) SIRT1 was knocked down with shRNA in HCT116 cells, subjected to heat shock at 42 °C for 1 h, and recovered at 37 °C for 2 h. Upper panel shows representative Western blots and lower panel shows quantification of ac-HDAC1 levels normalized to *β*-actin controls

The class III histone deacetylase SIRT1 also works as an important regulator for p53 activation network by deacetylating p53.^32, 33^ SIRT1 protein levels are affected by various oxidative stresses and diseases.^42, 43, 44^ Accordingly, we first investigated whether SIRT1 protein level is altered by heat-shock treatment. SIRT1 protein level is significantly reduced right after heat-shock treatment, and it remains low until 12 h after treatment (Figure 4a). We hypothesize that the rapid decrease of SIRT1 level may be due to the reduction of SIRT1 protein stability. To test this, HCT116 cells were treated with the proteasome inhibitor MG132 for 2 h before heat shock. The treatment prevents the reduction of SIRT1 level by heat shock (Figure 4b). In contrast, treatment with cycloheximide (CHX), a protein synthesis inhibitor, does not prevent the reduction of SIRT1 protein level after heat shock (Figure 4c). These results indicate that the decrease of SIRT1 protein level after heat shock is owing to the increase of SIRT1 protein degradation instead of the decrease of SIRT1 protein synthesis or gene expression.

It has recently been reported that during DNA double-strand breaks, SIRT1 deacetylates HDAC1 and the recruitment of active HDAC1 is necessary for the non-homologous end-joining repair pathway.^13^ Our data here show that the decrease of SIRT1 protein levels is associated with the increase of HDAC1 acetylation (Figures 1a–d) during heat shock, suggesting that SIRT1 may also regulate HDAC1 during heat-shock stress. The increase of HDAC1 acetylation was inhibited after heat-shock treatment in SIRT1-overexpressing cells, indicating that SIRT1 can deacetylate HDAC1 (Figure 4d). SIRT1 is then further knocked down by shRNA in HCT116 cells. The knock down of SIRT1 significantly increases HDAC1 acetylation levels (Figure 4e). Interestingly, the heat-shock treatment further enhances HDAC1 acetylation in SIRT1 knockdown cells (Figure 4e), indicating that the increase of HDAC1 acetylation after heat shock is not only due to the decrease of SIRT1-mediated deacetylation but also the increase of acetylation. These data suggest that SIRT1 regulates p53 and HDAC1 acetylation dynamically before and after heat-shock treatment. The increase of acetylated HDAC1 after heat shock may be regulated by both the increase of active acetylation and the decrease of deacetylation.

### P53 recruits acetylated HDAC1 to target genes in response to cell stress

**Figure 5:**
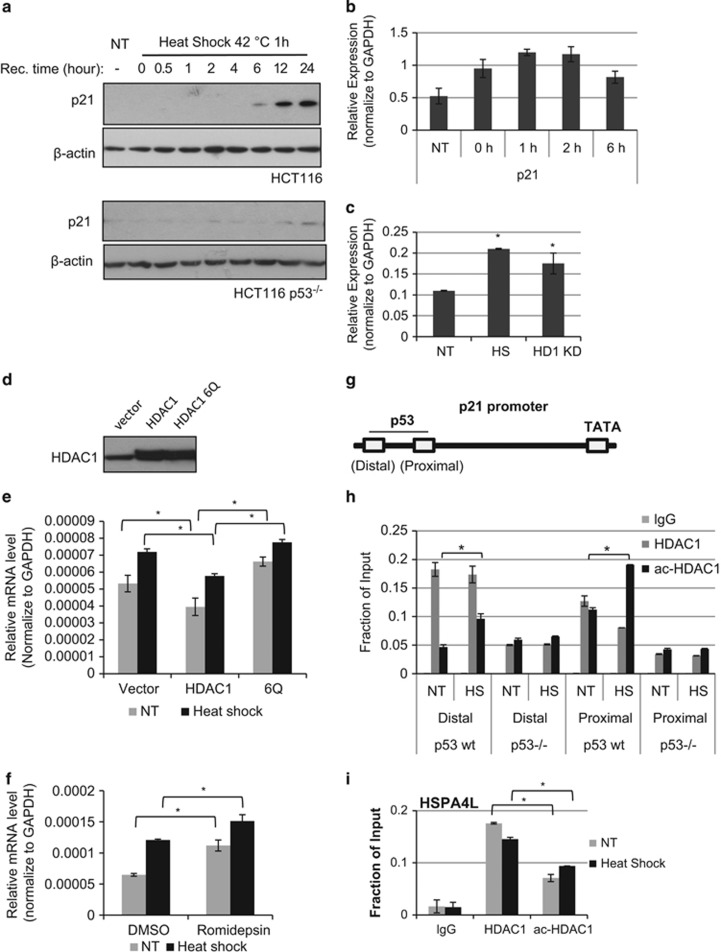
Dynamic HDAC1 acetylation regulates p21 promoter activity in response to cell stress. (**a**) HCT116 cells or p53 null HCT116 cells were subjected to heat shock at 42 °C for 1 h and then recovered at 37 °C for an indicated time period. P21 protein levels were determined by western blot. (**b**) HCT 116 cells were subjected to heat shock for 1 h and recovered for indicated period of time. p21 mRNA level was tested by RT qPCR. (**c**) P21 mRNA level was tested by RT-qPCR from HCT116 extracts with heat-shock treatment or HDAC1 knock down. The heat shock was performed at 42 °C for 1 h and then recovered at 37 °C for 8 h. P21 mRNA expression is shown relative to glyceraldehyde 3-phosphate dehydrogenase (GAPDH) (mean±S.E.). *Significant difference compared to the non-treatment (NT) cells (*P*<0.05 by Student's *t*-test). (**d**) HDAC1 and acetyl-mimic mutant 6Q were transfected into HCT116 cells and HDAC1 levels were determined by Western blot. (**e**) The p21 expression level was determined from cells overexpressed with HDAC1 or 6Q and treated with heat shock. The relative p21 levels were normalized to GAPDH (mean± S.E.). The extracts were prepared 8 h after recovered at 37 °C. *Significant difference compared with vector control cells (*P*<0.05 by Student's *t*-test). (**f**) HCT116 cells were treated by 20 *μ*M Romidepsin or DMSO and then subjected to heat shock. After 8 h of recovery, the p21 mRNA level was tested by RT-qPCR. The relative p21 levels were normalized to GAPHD (mean±S.E.). NT, non-treatment control compared with heat-shock treatment. *Significant difference compared with DMSO NT control cells (*P*<0.05 by Student's *t*-test). (**g**) P21 enhancer region with marked two p53 binding sites and TATA region. (**h**) ChIP assay for HDAC1 and acetylated HDAC1 in HCT116 wild-type and p53-null cells. The cells were subjected to heat shock at 42 °C for 1 h and then recovered at 37 °C for 2 h. The precipitated DNA was subjected to real-time PCR with primers amplifying p21 enhancer regions. *Significant difference compared with untreated control (*P*<0.05 by Student's *t*-test). (**i**) ChIP assay for HDAC1 and acetylated HDAC1 in HCT116 cells. The cells were subjected to heat shock at 42 °C for 1 h and then recovered at 37 °C for 2 h. The precipitated DNA was subjected to real-time PCR with primers amplifying HSPA4L gene promoter regions. *Significant difference (*P*<0.05 by Student's *t*-test)

P53 induces cellular response by activating target genes. One of the first target genes of p53 identified is the CDK inhibitor p21^Waf1/Cip1^.^17^ HDAC1 is also an important negative regulator for p21 gene expression.^45, 46, 47^ We investigated whether acetylated HDAC1 affects p21 gene expression. Heat-shock stress promotes the expression of p21 both in gene transcription and protein levels (Figures 5a and b). Importantly, the induction of p21 by heat shock is p53 dependent, as heat shock does not significantly induce p21 protein level in p53-null HCT116 cells (Figure 5a). The knock down of HDAC1 upregulates p21 expression, as expected (Figure 5c). HDAC1 or acetyl-mimic HDAC1 (6Q) were then overexpressed in HCT116 cells (Figure 5d). Overexpression of HDAC1 represses p21 expression in both untreated and heat-shock treated cells (Figure 5e); in contrast, acetyl-mimic HDAC1 increases p21 expression in both conditions (Figure 5e). The treatment of Romidepsin, a specific inhibitor for HDAC1/2, also elevates p21 expression (Figure 5f). These data indicate that the acetylation of HDAC1 after heat shock contributes to an increase in p21 gene expression.

Next, we investigated whether acetylated HDAC1 regulates p21 gene expression through p53 binding sites at the p21 promoter. There are two p53 binding sites at the p21 enhancer region, the distal binding site and the proximal binding site (Figure 5g; Lagger *et al.*,^47^ Lin *et al.*,^48^ Gui *et al.*^49^). Chromatin immunoprecipitation (ChIP) analysis was performed with HDAC1 and acetyl-HDAC1 antibodies. HDAC1 binding does not significantly change, but acetyl-HDAC1 binding increased at the distal site (Figure 5h). At the proximal p53 binding site, HDAC1 binding was reduced after the heat shock and acetylated HDAC1 binding increased (Figure 5h). These results indicate that HDAC1 deacetylase activity at both the p53 binding site decreases after heat shock. In contrast, p53-null cells recruit low levels of HDAC1 and acetylated HDAC1 and their binding levels do not change after heat shock (Figure 5h), indicating the dynamic recruitment of HDAC1 and acetylated HDAC1 before and after heat-shock treatment is p53 dependent. *HSPA4L* is another gene whose promoter activity is regulated by p53 and HDAC1.^50^ Consistent with HDAC1 recruitment patterns at the p21 enhancer region, HDAC1 binding decreased and acetyl-HDAC1 recruitment increased at the p53 binding site of the HSPA4L enhancer (Figure 5i).

**Figure 6:**
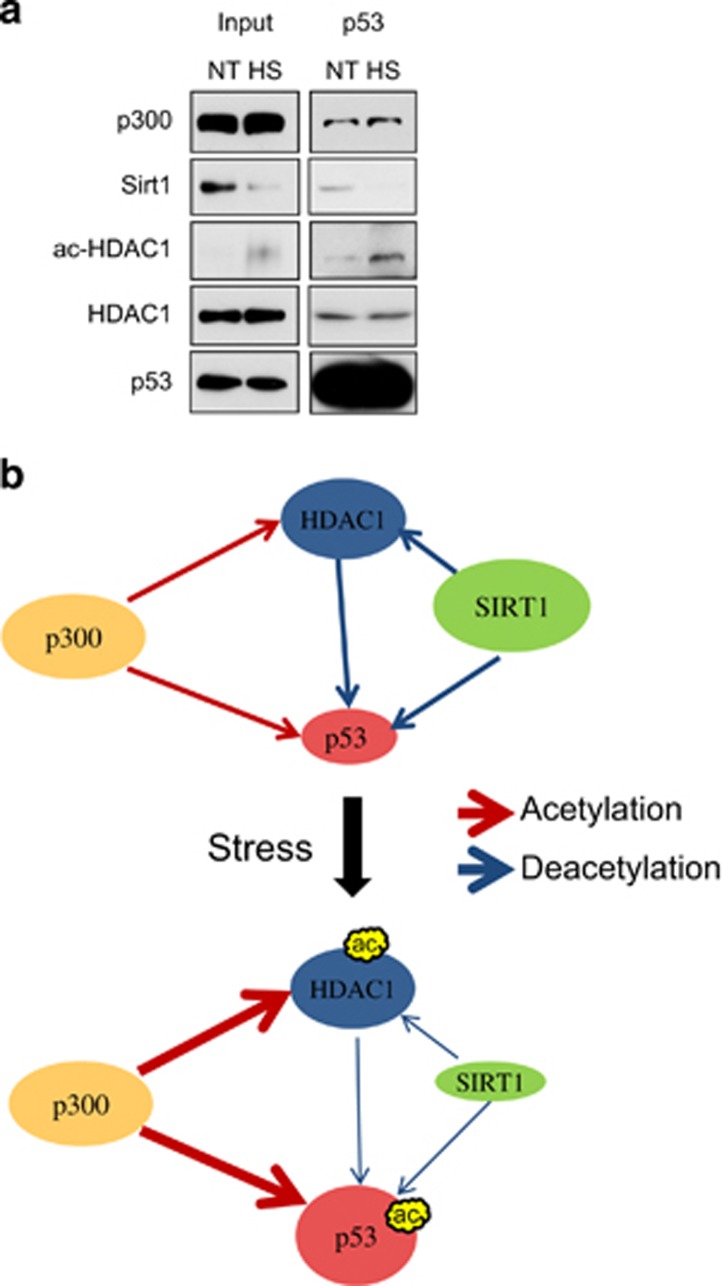
P53 interacts with acetylated HDAC1 after heat stress. (**a**) HCT116 cells were treated with heat shock at 42 °C for 1 h and then recovered at 37 °C for 2 h. After treatment, the cells were subjected to immunoprecipitation by using the p53 antibody. The p300, SIRT1, ac-HDAC1 and HDAC1 levels were examined by western blotting. (**b**) Model depicting the dynamic regulation of p53 and HDAC1 acetylation upon cellular stress

To further elucidate if p53 physically recruits acetylated HDAC1, HCT116 cells with or without heat-shock treatment were immunoprecipitated with the p53 antibody. P53-associated HDAC1 does not change significantly; however, p53-associated acetylated HDAC1 increases markedly, indicating that p53-associated deacetylase activity decreases after heat shock (Figure 6a). P53-associated SIRT1 level also decreases, consistent with the notion that SIRT1 protein level reduces after heat shock (Figures 4a and 6a). Interestingly, the association of p300 with p53 does not change significantly, suggesting that the reduction of deacetylase activity may have a dominant role in upregulating p53 acetylation during stress response.

## Discussion

The p53 tumor suppressor is a tightly regulated protein that has an essential role in preventing cell-cycle progression and promoting apoptosis when encountering cellular stress. Having a short half-life, p53 is normally maintained at a low level in unstressed mammalian cells by continuous ubiquitination and subsequent 26S proteasome-mediated degradation. When the cell is confronted with stress, p53 ubiquitination is suppressed and, instead, acetylation of p53 increases. These effects result in the activation of the downstream p53 target genes, most of which are associated with the regulation of cell-cycle arrest, apoptosis and/or DNA repair processes, processes that function to prevent the proliferation of damaged cells.^26, 27, 28, 51^ The increase of p53 acetylation can be achieved by rebalancing the acetylation and deacetylation. Our study suggests that the reduction of deacetylation is a major key for the increase of p53 acetylation level in stressed cells. There are three major events that are involved in enhancing p53 acetylation during stress. First, p53-bound HDAC1 is acetylated by p300 under stress, which reduces the catalytic activity of HDAC1, and therefore reduces p53 deacetylation. Second, SIRT1, another major deacetylase for p53, is destabilized and degraded under proteasome-dependent pathways, and this process further reduces p53 deacetylation. Third, decreased SIRT1 level induces the reduction of deacetylation of acetylated HDAC1, which eventually results in the reduction of p53 deacetylation (Figure 6b).

SIRT1 was considered as both a tumor suppressor and a tumor promoter.^52^ SIRT1 inhibits expression and/or the activity of several oncogenes, leading to reduced cell proliferation, increased apoptosis and tumor suppression.^53, 54^ In response to DNA damage, SIRT1 promotes DNA repair and maintains genome integrity.^13, 33, 55^ However, on the other hand, SIRT1 protein is overexpressed in many cancer types.^52^ More importantly, SIRT1 inactivates p53, leading to the downregulation of p53-mediated growth arrest and apoptosis,^31, 32, 33, 34^ which results in an increased risk of cancer. SIRT1 protein level is dynamically regulated in response to stress. It has been shown that exposure to oxidants/aldehydes promotes SIRT1 protein degradation.^42^ SIRT1 is ubiquitinated and degraded in response to insulin and this process is JNK1 phosphorylation dependent on serine 47.^56^ We found that SIRT1 is rapidly depleted upon heat shock and the degradation of SIRT1 is proteasome dependent. It remains to be determined whether phosphorylation on serine 47 is also required for SIRT1 degradation upon heat shock.

HDAC1 acetylation is induced in several physiological events.^11, 13, 14^ Increased HDAC1 acetylation reduces HDAC1-associated deacetylase activity, and therefore directly affects target gene transcription. However, how HDAC1 acetylation is induced in response to cellular signal is still not understood. Our study suggests that the increase of p300-mediated acetylation, and the decrease of SIRT1-mediated deacetylation, are key events for the increase of HDAC1 acetylation in response to cellular stress. It remains to be further investigated whether HDAC1 acetylation, whose modification negatively regulates HDAC1-associated corepressor complex activity, is downregulated in cancer models.

## Materials and Methods

### Cell culture and stress treatment

The HCT116, HCT116 p53^−/−^, HT-29 and MCF-7 cells were grown at 37 °C in a 5% CO_2_ atmosphere in DMEM medium containing 10% fetal bovine serum. Heat shock of cultured cells was performed by incubating cells in a 42 °C tissue culture incubator for an indicated time and then putting back to 37 °C for recovery. Control cells were maintained in normal conditions. For H_2_O_2_ treatment, HCT116 cells were treated with 100 *μ*M H_2_O_2_ for an indicated time. UV treatment of cells (80 J/m^2^ UVC) was carried out with Stratalinker 2400 (Stratagene, La Jolla, CA, USA).

### Stable cell lines

HDAC1, HDAC1 6R or HDAC1 6Q was stably overexpressed in HCT116 cells using the retroviral vector pOZ with Flag tag sequences.^57, 58^ The pOZ vector coexpresses the IL-2 receptor, which is located at the cell membrane to facilitate the purification of transfected cells by sorting with IL-2R antibodies coupled to magnetic beads. The pOZ plasmid containing wild-type or mutant HDAC1 coding sequences were transfected into the packaging cell line Phoenix A. The retrovirus particles were harvested and used to infect HCT116 cells. The cells were sorted for IL-2R expression. Stable clones expressing moderate levels of HDAC1 or HDAC1 mutants were selected.

### Real-time quantitative RT-PCR

Total cellular RNA was isolated from 1 × 10^5^ cells and reverse transcribed into cDNA using SuperScript reverse transcriptase and oligo (dT) primers (Invitrogen, Carlsbad, CA). The real-time PCR was performed using Power SYBR Green PCR Master Mix (Bio-Rad, Hercules, CA, USA). The p21 primer sequences are as follows: forward, 5′-GTGGACCTGTCACTGTCTT-3′ reverse, 5′-GCGTTTGGAGTGGTAGAAATC-3′.

### Antibodies

Antibodies for western, immunoprecipitation and ChIP are as follows: anti-HDAC1 (Pierce, Rockford, IL, USA), anti-SIRT1 (Cell Signaling Technology, Danvers, MA, USA), anti-p53 (Cell Signaling Technology), anti-p300 (Santa Cruz Biotechnology, Santa Cruz, CA, usa), anti-CBP (Cell Signaling Technology), anti-PCAF (Santa Cruz Biotechnology), anti-p21 (Cell Signaling Technology), anti-PARP (Cell Signaling Technology), anti-ac373p53 (Cell Signaling Technology), anti-ac382 p53 (Cell Signaling Technology), anti-acetylated HDAC1^14^ and anti-*β*-actin (Sigma-Aldrich, St. Louis, MO, USA).

### ChIP assay

ChIP assay was performed as described previously.^11^ Briefly, 5 × 10^6^ HCT116 cells treated with indicated stress were subjected to formaldehyde crosslink. Cells were sonicated to obtain chromatin fragments of size ranging between 300 and 500 bp. The crosslinked chromatin was subsequently immunoprecipitated with indicated antibodies or normal rabbit lgG as a control. The purified DNA from precipitated chromatin was subjected to qPCR amplification. The primers for ChIP are as follows: p21 – 2392 site (FP, 5′-TGCTTCCCAGGAACATGCTTG-3′ RP, 5′-CTGAAAACAGGCAGCCCAAGG-3′), p21 – 1440 site (FP, 5′-GCAGAGGAGAAAGAAGCC TG-3′ RP, 5′-GCAGAGGATGGATTGTTCATC-3′) and *HSPA4L* gene (FP, 5′-TGCCAAAACAACCCAAAAATG-3′ RP, 5′-AATGGAGGCTGCTGAGCTATC-3′).

### Plasmid transfection and shRNA knockdown

Flag-pCDNA3.1-HDAC1 and Flag-pCDNA3.1-SIRT1 were obtained from Dr. Ed Seto at the Moffitt Cancer Center (Tampa, FL, USA). The plasmids were transfected into HCT116 cells using Lipofectamine 2000 according to the manufacturer's protocol (Invitrogen). Human CBP, PCAF, p300 and SIRT1 shRNAs were obtained from the TRC shRNA library (Open Biosystems, Thermo Fisher Scientific, Logan, MA, USA). The lentiviral particles were generated according to the manufacturer's instruction. HCT116 cells were infected with the virus and selected in DMEM medium containing 1 *μ*g/ml puromycin one day after infection. The RNAi consortium numbers (TRCNS) are as follows: shSIRT1 (TRCN0000018981), shp300 (TRCN0000039883), shCBP (TRCN0000006487) and shPCAF (TRCN00000018531).

### *In vitro* acetylation and deacetylase assay

Flag-HDAC1 and GST-p53 were first acetylated by Flag-p300 as described previously.^11^ The samples were then dialyzed against 1 liter of HAT buffer (50 mM Tris-HCl (pH 8.0), 10% glycerol, 1 mM DTT, 1 mM PMSF, 0.1 mM EDTA, 10 mM butyrate) for 1 h at 4 °C before the subsequent deacetylase assay. Acetylated p53 was incubated with acetylated or control HDAC1 in HDAC assay buffer (20 mM Tris-HCl (pH 8.0), 150 mM NaCl, 5% glycerol, 0.5 mM EDTA). The products were then subjected to western blot with indicated antibodies.

To test HDAC1 activity, deacetylation assay of purified HDAC1 and stress-treated HDAC1 were carried out by mixing HDAC1 and ^3^H-labeled acetylated histones in 50 *μ*l assay buffer (20 mM Tris-HCl (pH 8.0), 150 mM NaCl, 5% glycerol, 0.5 mM EDTA). The reaction was stopped by adding 50 *μ*l of stop buffer (1.44 M HCl, 0.24 M HOAc). ^3^H-labeled acetate was extracted with ethyl acetate. After centrifugation, the upper organic phase was quantified by a liquid scintillation counter.

### Protein purification from baculovirus

Flag-HDAC1 and p300-expressing baculovirus vectors have been described.^11^ The sf9 cells were infected with recombinant baculovirus. The infected cells were collected at 48 h after infection and proteins were purified by anti-Flag-conjugated M2 agarose (Sigma, St. Louis, MO, USA). The Flag-tagged proteins were then eluted in the lysis buffer (20 mM Tris-HCl (pH 8.0), 10% glycerol, 5 mM MgCl_2_, 150 mM KCl, 0.1% Tween-20) supplemented with 200 *μ*g/ml of Flag peptide (Sigma). The GST-tagged p53 protein was purchased from EMD Millipore Corporation (Bedford, MA, USA).

## Abbreviations

HAT: histone acetyltransferase
HDAC: histone deacetylase
SIRT1: sirtuin 1
ChIP: chromatin immunoprecipitation
CHX: cycloheximide

## References

[bib1] Choudhary C, Kumar C, Gnad F, Nielsen ML, Rehman M, Walther TC et al. Lysine acetylation targets protein complexes and co-regulates major cellular functions. Science 2009; 325: 834–840. 19608861 10.1126/science.1175371

[bib2] Cress WD, Seto E. Histone deacetylases, transcriptional control, and cancer. J Cell Physiol 2000; 184: 1–16. 10825229 10.1002/(SICI)1097-4652(200007)184:1<1::AID-JCP1>3.0.CO;2-7

[bib3] Blander G, Guarente L. The Sir2 family of protein deacetylases. Annu Rev Biochem 2004; 73: 417–435. 15189148 10.1146/annurev.biochem.73.011303.073651

[bib4] Haigis MC, Guarente LP. Mammalian sirtuins – emerging roles in physiology, aging, and calorie restriction. Genes Dev 2006; 20: 2913–2921. 17079682 10.1101/gad.1467506

[bib5] Saunders LR, Verdin E. Sirtuins: critical regulators at the crossroads between cancer and aging. Oncogene 2007; 26: 5489–5504. 17694089 10.1038/sj.onc.1210616

[bib6] Cho Y, Griswold A, Campbell C, Min KT. Individual histone deacetylases in *Drosophila* modulate transcription of distinct genes. Genomics 2005; 86: 606–617. 16137856 10.1016/j.ygeno.2005.07.007

[bib7] Foglietti C, Filocamo G, Cundari E, De Rinaldis E, Lahm A, Cortese R et al. Dissecting the biological functions of *Drosophila* histone deacetylases by RNA interference and transcriptional profiling. J Biol Chem 2006; 281: 17968–17976. 16632473 10.1074/jbc.M511945200

[bib8] Yang XJ, Seto E. The Rpd3/Hda1 family of lysine deacetylases: from bacteria and yeast to mice and men. Nat Rev Mol Cell Biol 2008; 9: 206–218. 18292778 10.1038/nrm2346 PMC2667380

[bib9] Segre CV, Chiocca S. Regulating the regulators: the post-translational code of class I HDAC1 and HDAC2. J Biomed Biotechnol 2011; 2011: 690848. 21197454 10.1155/2011/690848 PMC3004424

[bib10] Osoata GO, Yamamura S, Ito M, Vuppusetty C, Adcock IM, Barnes PJ et al. Nitration of distinct tyrosine residues causes inactivation of histone deacetylase 2. Biochem Biophys Res Commun 2009; 384: 366–371. 19410558 10.1016/j.bbrc.2009.04.128

[bib11] Qiu Y, Zhao Y, Becker M, John S, Parekh BS, Huang S et al. HDAC1 acetylation is linked to progressive modulation of steroid receptor-induced gene transcription. Mol Cell 2006; 22: 669–679. 16762839 10.1016/j.molcel.2006.04.019

[bib12] Oh YM, Kwon YE, Kim JM, Bae SJ, Lee BK, Yoo SJ et al. Chfr is linked to tumour metastasis through the downregulation of HDAC1. Nat Cell Biol 2009; 11: 295–302. 19182791 10.1038/ncb1837

[bib13] Dobbin MM, Madabhushi R, Pan L, Chen Y, Kim D, Gao J et al. SIRT1 collaborates with ATM and HDAC1 to maintain genomic stability in neurons. Nat Neurosci 2013; 16: 1008–1015. 23852118 10.1038/nn.3460 PMC4758134

[bib14] Yang T, Jian W, Luo Y, Fu X, Noguchi C, Bungert J et al. Acetylation of histone deacetylase 1 regulates NuRD corepressor complex activity. J Biol Chem 2012; 287: 40279–40291. 23014989 10.1074/jbc.M112.349704 PMC3504744

[bib15] Luo Y, Jian W, Stavreva D, Fu X, Hager G, Bungert J et al. *Trans*-regulation of histone deacetylase activities through acetylation. J Biol Chem 2009; 284: 34901–34910. 19822520 10.1074/jbc.M109.038356 PMC2787352

[bib16] Vogelstein B, Lane D, Levine AJ. Surfing the p53 network. Nature 2000; 408: 307–310. 11099028 10.1038/35042675

[bib17] el-Deiry WS, Harper JW, O'Connor PM, Velculescu VE, Canman CE, Jackman J et al. WAF1/CIP1 is induced in p53-mediated G1 arrest and apoptosis. Cancer Res 1994; 54: 1169–1174. 8118801

[bib18] Sherr CJ, Roberts JM. CDK inhibitors: positive and negative regulators of G1-phase progression. Genes Dev 1999; 13: 1501–1512. 10385618 10.1101/gad.13.12.1501

[bib19] Kastan MB, Onyekwere O, Sidransky D, Vogelstein B, Craig RW. Participation of p53 protein in the cellular response to DNA damage. Cancer Res 1991; 51: 6304–6311. 1933891

[bib20] Ito A, Lai CH, Zhao X, Saito S, Hamilton MH, Appella E et al. P300/CBP-mediated p53 acetylation is commonly induced by p53-activating agents and inhibited by MDM2. EMBO J 2001; 20: 1331–1340. 11250899 10.1093/emboj/20.6.1331 PMC145533

[bib21] Gu W, Roeder RG. Activation of p53 sequence-specific DNA binding by acetylation of the p53 C-terminal domain. Cell 1997; 90: 595–606. 9288740 10.1016/s0092-8674(00)80521-8

[bib22] Sykes SM, Mellert HS, Holbert MA, Li K, Marmorstein R, Lane WS et al. Acetylation of the p53 DNA-binding domain regulates apoptosis induction. Mol Cell 2006; 24: 841–851. 17189187 10.1016/j.molcel.2006.11.026 PMC1766330

[bib23] Kruse JP, Gu W. SnapShot: p53 posttranslational modifications. Cell 2008; 133: 930–930 e931. 18510935 10.1016/j.cell.2008.05.020 PMC3690516

[bib24] Dai C, Gu W. P53 post-translational modification: deregulated in tumorigenesis. Trends Mol Med 2010; 16: 528–536. 20932800 10.1016/j.molmed.2010.09.002 PMC2978905

[bib25] Ito A, Kawaguchi Y, Lai CH, Kovacs JJ, Higashimoto Y, Appella E et al. MDM2-HDAC1-mediated deacetylation of p53 is required for its degradation. EMBO J 2002; 21: 6236–6245. 12426395 10.1093/emboj/cdf616 PMC137207

[bib26] Brooks CL, Gu W. The impact of acetylation and deacetylation on the p53 pathway. Protein Cell 2011; 2: 456–462. 21748595 10.1007/s13238-011-1063-9 PMC3690542

[bib27] Bode AM, Dong Z. Post-translational modification of p53 in tumorigenesis. Nat Rev Cancer 2004; 4: 793–805. 15510160 10.1038/nrc1455

[bib28] Brooks CL, Gu W. Ubiquitination, phosphorylation and acetylation: the molecular basis for p53 regulation. Curr Opin Cell Biol 2003; 15: 164–171. 12648672 10.1016/s0955-0674(03)00003-6

[bib29] Lee JT, Gu W. SIRT1: regulator of p53 deacetylation. Genes Cancer 2013; 4: 112–117. 24020002 10.1177/1947601913484496 PMC3764473

[bib30] Luo J, Su F, Chen D, Shiloh A, Gu W. Deacetylation of p53 modulates its effect on cell growth and apoptosis. Nature 2000; 408: 377–381. 11099047 10.1038/35042612

[bib31] Luo J, Nikolaev AY, Imai S, Chen D, Su F, Shiloh A et al. Negative control of p53 by Sir2alpha promotes cell survival under stress. Cell 2001; 107: 137–148. 11672522 10.1016/s0092-8674(01)00524-4

[bib32] Vaziri H, Dessain SK, Ng Eaton E, Imai SI, Frye RA, Pandita TK et al. hSIR2(SIRT1) functions as an NAD-dependent p53 deacetylase. Cell 2001; 107: 149–159. 11672523 10.1016/s0092-8674(01)00527-x

[bib33] Cheng HL, Mostoslavsky R, Saito S, Manis JP, Gu Y, Patel P et al. Developmental defects and p53 hyperacetylation in Sir2 homolog (SIRT1)-deficient mice. Proc Natl Acad Sci USA 2003; 100: 10794–10799. 12960381 10.1073/pnas.1934713100 PMC196882

[bib34] Lain S, Hollick JJ, Campbell J, Staples OD, Higgins M, Aoubala M et al. Discovery, *in vivo* activity, and mechanism of action of a small-molecule p53 activator. Cancer Cell 2008; 13: 454–463. 18455128 10.1016/j.ccr.2008.03.004 PMC2742717

[bib35] Nitta M, Okamura H, Aizawa S, Yamaizumi M. Heat shock induces transient p53-dependent cell cycle arrest at G1/S. Oncogene 1997; 15: 561–568. 9247309 10.1038/sj.onc.1201210

[bib36] Matsumoto H, Shimura M, Omatsu T, Okaichi K, Majima H, Ohnishi T. P53 proteins accumulated by heat stress associate with heat shock proteins HSP72/HSC73 in human glioblastoma cell lines. Cancer Lett 1994; 87: 39–46. 7954368 10.1016/0304-3835(94)90407-3

[bib37] Nagashima M, Shiseki M, Miura K, Hagiwara K, Linke SP, Pedeux R et al. DNA damage-inducible gene p33ING2 negatively regulates cell proliferation through acetylation of p53. Proc Natl Acad Sci USA 2001; 98: 9671–9676. 11481424 10.1073/pnas.161151798 PMC55510

[bib38] Sakaguchi K, Herrera JE, Saito S, Miki T, Bustin M, Vassilev A et al. DNA damage activates p53 through a phosphorylation-acetylation cascade. Genes Dev 1998; 12: 2831–2841. 9744860 10.1101/gad.12.18.2831 PMC317174

[bib39] Ito A, Kawaguchi Y, Lai C-H, Kovacs JJ, Higashimoto Y, Appella E et al. MDM2–HDAC1-mediated deacetylation of p53 is required for its degradation. EMBO J 2002; 21: 6236–6245. 12426395 10.1093/emboj/cdf616 PMC137207

[bib40] Yang H, Salz T, Zajac-Kaye M, Liao D, Huang S, Qiu Y. Overexpression of histone deacetylases in cancer cells is controlled by interplay of transcription factors and epigenetic modulators. FASEB J 2014; 28: 4265–4279. 24948597 10.1096/fj.14-250654 PMC4202103

[bib41] Yang H, Pinello CE, Luo J, Li D, Wang Y, Zhao LY et al. Small-molecule inhibitors of acetyltransferase p300 identified by high-throughput screening are potent anticancer agents. Mol Cancer Ther 2013; 12: 610–620. 23625935 10.1158/1535-7163.MCT-12-0930 PMC3651759

[bib42] Caito S, Rajendrasozhan S, Cook S, Chung S, Yao H, Friedman AE et al. SIRT1 is a redox-sensitive deacetylase that is post-translationally modified by oxidants and carbonyl stress. FASEB J 2010; 24: 3145–3159. 20385619 10.1096/fj.09-151308 PMC2923349

[bib43] Kwon HS, Ott M. The ups and downs of SIRT1. Trends Biochem Sci 2008; 33: 517–525. 18805010 10.1016/j.tibs.2008.08.001

[bib44] Yang Y, Fu W, Chen J, Olashaw N, Zhang X, Nicosia SV et al. SIRT1 sumoylation regulates its deacetylase activity and cellular response to genotoxic stress. Nat Cell Biol 2007; 9: 1253–1262. 17934453 10.1038/ncb1645 PMC3201724

[bib45] Zupkovitz G, Grausenburger R, Brunmeir R, Senese S, Tischler J, Jurkin J et al. The cyclin-dependent kinase inhibitor p21 is a crucial target for histone deacetylase 1 as a regulator of cellular proliferation. Mol Cell Biol 2010; 30: 1171–1181. 20028735 10.1128/MCB.01500-09 PMC2820891

[bib46] Yamaguchi T, Cubizolles F, Zhang Y, Reichert N, Kohler H, Seiser C et al. Histone deacetylases 1 and 2 act in concert to promote the G1-to-S progression. Genes Dev 2010; 24: 455–469. 20194438 10.1101/gad.552310 PMC2827841

[bib47] Lagger G, Doetzlhofer A, Schuettengruber B, Haidweger E, Simboeck E, Tischler J et al. The tumor suppressor p53 and histone deacetylase 1 are antagonistic regulators of the cyclin-dependent kinase inhibitor p21/WAF1/CIP1 gene. Mol Cell Biol 2003; 23: 2669–2679. 12665570 10.1128/MCB.23.8.2669-2679.2003 PMC152549

[bib48] Lin YC, Lin JH, Chou CW, Chang YF, Yeh SH, Chen CC. Statins increase p21 through inhibition of histone deacetylase activity and release of promoter-associated HDAC1/2. Cancer Res 2008; 68: 2375–2383. 18381445 10.1158/0008-5472.CAN-07-5807

[bib49] Gui CY, Ngo L, Xu WS, Richon VM, Marks PA. Histone deacetylase (HDAC) inhibitor activation of p21WAF1 involves changes in promoter-associated proteins, including HDAC1. Proc Natl Acad Sci USA 2004; 101: 1241–1246. 14734806 10.1073/pnas.0307708100 PMC337037

[bib50] Ram O, Goren A, Amit I, Shoresh N, Yosef N, Ernst J et al. Combinatorial patterning of chromatin regulators uncovered by genome-wide location analysis in human cells. Cell 2011; 147: 1628–1639. 22196736 10.1016/j.cell.2011.09.057 PMC3312319

[bib51] Brooks CL, Gu W. New insights into p53 activation. Cell Res 2010; 20: 614–621. 20404858 10.1038/cr.2010.53 PMC3070262

[bib52] Deng CX. SIRT1, is it a tumor promoter or tumor suppressor? Int J Biol Sci 2009; 5: 147–152. 19173036 10.7150/ijbs.5.147 PMC2631220

[bib53] Yeung F, Hoberg JE, Ramsey CS, Keller MD, Jones DR, Frye RA et al. Modulation of NF-kappaB-dependent transcription and cell survival by the SIRT1 deacetylase. EMBO J 2004; 23: 2369–2380. 15152190 10.1038/sj.emboj.7600244 PMC423286

[bib54] Wang RH, Zheng Y, Kim HS, Xu X, Cao L, Luhasen T et al. Interplay among BRCA1, SIRT1, and Survivin during BRCA1-associated tumorigenesis. Mol Cell 2008; 32: 11–20. 18851829 10.1016/j.molcel.2008.09.011 PMC2577018

[bib55] Wang RH, Sengupta K, Li C, Kim HS, Cao L, Xiao C et al. Impaired DNA damage response, genome instability, and tumorigenesis in SIRT1 mutant mice. Cancer Cell 2008; 14: 312–323. 18835033 10.1016/j.ccr.2008.09.001 PMC2643030

[bib56] Gao Z, Zhang J, Kheterpal I, Kennedy N, Davis RJ, Ye J. Sirtuin 1 (SIRT1) protein degradation in response to persistent c-Jun N-terminal kinase 1 (JNK1) activation contributes to hepatic steatosis in obesity. J Biol Chem 2011; 286: 22227–22234. 21540183 10.1074/jbc.M111.228874 PMC3121368

[bib57] Humphrey GW, Wang Y, Russanova VR, Hirai T, Qin J, Nakatani Y et al. Stable histone deacetylase complexes distinguished by the presence of SANT domain proteins CoREST/kiaa0071 and Mta-L1. J Biol Chem 2001; 276: 6817–6824. 11102443 10.1074/jbc.M007372200

[bib58] Nakatani Y, Ogryzko V. Immunoaffinity purification of mammalian protein complexes. Methods Enzymol 2003; 370: 430–444. 14712665 10.1016/S0076-6879(03)70037-8

